# Factors influencing physical activity behavior in older adults with subjective cognitive decline: an empirical study using SEM and fsQCA methods

**DOI:** 10.3389/fpubh.2024.1409614

**Published:** 2024-10-17

**Authors:** Wei Li, Sixue Hong, Yiping Chen, Yang Zhao, Limei Wang

**Affiliations:** ^1^International Medical Service Department, Peking Union Medical College Hospital (CAMS), Beijing, China; ^2^School of Nursing, Shanxi Medical University, Taiyuan, China

**Keywords:** physical activity, older adults, subjective cognitive decline, factors, fsQCA

## Abstract

**Objectives:**

Despite the evident potential benefits of engaging in physical activity (PA) for older adults with subjective cognitive decline (SCD), their PA levels remains low. Previous research has predominantly focused on PA behaviors in individuals with dementia/mild cognitive impairment, with limited attention given to those with SCD. Therefore, this study aims to identify key factors influencing PA behavior in older adults with SCD based on the Self-Determination Theory (SDT) and the capability-opportunity-motivation (COM-B) model.

**Methods:**

Three hundred and three individuals aged 60 and above with SCD participated in this study. A face-to-face structured questionnaire survey was conducted. Data were analyzed using Structural Equation Modeling (SEM) and Fuzzy Set Qualitative Comparative Analysis (fsQCA).

**Results:**

SEM results indicate that PA social support primarily influences PA behavior through three indirect pathways: the separate mediating effect of basic psychological needs, the separate mediating effect of motivation, and the chained mediating effect of both. Physical literacy, on the other hand, influences PA behavior through the separate mediating effect of motivation. Necessary conditions analysis by fsQCA reveals that no single factor is necessary for promoting PA behavior in older adults with SCD, while sufficiency analysis identifies four different combinations of factors leading to PA behavior.

**Conclusion:**

The model derived from the framework of SDT and the COM-B model effectively explains and predicts the interrelationships among variables. Physical activity behavior in older individuals with SCD is the result of multifactorial synergies.

## Introduction

Alzheimer’s disease (AD) is a common neurodegenerative disease that primarily affects the older adult and is a major cause of dementia. With the global aging population, the prevalence of AD is gradually increasing (1). Currently, AD dementia ranks among the top 10 global causes of death, yet there are no reliable methods for preventing, slowing down, or curing this disease. The costs associated with AD dementia almost exceed those of any other disease, with over 80% of these costs being covered by social care services and informal caregiving, posing a serious public health issue (2). Therefore, early intervention in the preclinical stages of AD dementia to modify or delay disease progression has become an urgent task for governments and researchers worldwide. Alzheimer’s disease is considered a gradual pathological process, divided into seven stages (Stages 0 to 6), reflecting the increasing severity of the disease. Stage 2 is characterized by subjective cognitive decline (SCD) (3), indicating the preclinical phase of AD dementia. In comparison to stage 1 (no cognitive impairment), Stage 2 involves mild neuronal damage, where cognitive function is not entirely normal but does not meet the criteria for mild cognitive impairment (MCI; Stage 3). During the SCD stage, individuals still maintain intact cognitive function and can employ compensatory mechanisms. Cognitive decline during this stage is reversible, making it the opportune time (as the secondary prevention) for prevention and intervention (4).

The prevalence of SCD among the older adult is significant. The Centers for Disease Control and Prevention (CDC) in the United States found that the prevalence of SCD is 9.9% among older adult individuals aged 64 to 74, and it increases to 14.3% among those aged 75 and older (5). A study conducted in China involving 2,689 participants revealed that 18.8% of adults aged 60 to 80 observed SCD (5). Increasingly, scholars advocate for early diagnosis and prevention, aiming to intervene as early as the preclinical stages of AD dementia. Therefore, SCD has become an important intervention target in the preclinical stages of AD dementia.

However, there are currently no effective drugs available to slow cognitive decline in older adult individuals with SCD. The World Health Organization’s “Guidelines on Risk Reduction of Cognitive Decline and Dementia” provide evidence-based intervention recommendations (6) to reduce modifiable risk factors for dementia, emphasizing that lack of physical activity is one of the significant risk factors. Evidence indicates that insufficient physical activity is the largest modifiable risk factor for dementia, accounting for 17.9% of the population-attributable risk. Physical activity is a promising non-pharmacological therapy and is the most cost-effective method among all modifiable risk factors. Increasing research has explored the role of physical activity in slowing cognitive decline (7). Although the effectiveness of physical activity in individuals with MCI and AD dementia remains controversial (8, 9), it has shown positive effects in the preclinical stages of MCI and AD dementia. Therefore, for older adult individuals with SCD, physical activity is now considered an effective method to reduce the risk of AD dementia.

However, older adults with SCD typically engage in less PA, and there is limited research specifically studying their PA behavior (10). Additionally, despite similarities in PA behavior between older adults with SCD and those with dementia/MCI, some known influencing factors in the latter group lack specific theoretical support (11), making it challenging to directly guide the PA behavior of older adults with SCD. Moreover, empirical research is less on the theoretical framework models of influencing factors for PA behavior in older adults with SCD, dementia, or MCI (12). In-depth exploration of the pathway mechanisms of PA behavior contributes to guiding community care practices and providing robust support to enhance the PA levels of older adults with SCD. In this study, we proposed a mediation model based on Self-Determination Theory (SDT) (13) and the Capability-Opportunity-Motivation (COM-B) model (14) to investigate factors influencing PA behavior in older adults with SCD. We included four variables—physical literacy, PA social support, basic psychological needs, and motivation—and explored their complex relationships with PA behavior. The aim was to fill the gap in the understanding of the influencing mechanisms of PA behavior in older adults with SCD through a comprehensive model.

## Background

Currently, there is limited research directly exploring PA in older adults with SCD. One contributing factor may be the vague conceptualization of SCD (15). Consequently, research has predominantly focused on populations with dementia/MCI. However, due to the specific characteristics of SCD patients, such as the vulnerability resulting from cognitive decline (16), there is a certain overlap in the study of PA behaviors between older adults with SCD and older adults with dementia/MCI. In the scarcity of research on PA behaviors in older adults with SCD, studies on the influencing factors of PA behaviors in dementia/MCI patients lay a solid theoretical foundation for understanding and promoting PA in older adults with SCD.

The COM-B model emphasizes the importance of the external environment on individual behavior. According to the COM-B model, Opportunity includes social and environmental influences. As a crucial pathway in the COM-B model, individuals are influenced by opportunities (Opportunity), leading to the generation of motivation (Motivation), and subsequently engaging in PA behavior (Behavior). Therefore, the impact of the external environment on individual PA behavior is significant. A recent umbrella review (11) highlighted environmental resources for PA and social influences (collectively referred to as the external environment) as the two highest-rated factors affecting older adults with dementia/MCI. Thus, assessing the external environment of PA in older adults with SCD is crucial, with PA social support as a key component reflecting perceived external environmental support, serving as a vital indicator for evaluation (17, 18). PA social support includes support from family and friends, as well as physical support for physical activities, such as instrumental and informational support. External support not only creates objective conditions for older adults with SCD to engage in PA but also fosters motivation internalization through emotional and action support, promoting active and sustained participation in PA (19). It can be inferred that the greater the PA social support, the stronger the motivation for older adults with SCD to engage in PA, leading to the realization of PA.

In the COM-B model, PA social support directly promotes motivation; however, the process of motivation generation may be complex. According to self-determination theory, there is an important mediating factor between social support and motivation, namely basic psychological needs. Basic psychological needs encompass autonomy, competence, and relatedness. PA social support, by satisfying any of these basic psychological needs, leads to the internalization of external motivation and the reinforcement of intrinsic motivation, thereby promoting PA. Previous studies have consistently indicated that basic psychological needs not only promote the formation of PA behavior through motivation but also directly impact PA behavior. Therefore, the introduction of basic psychological needs variables can better explain how PA social support stimulates motivation, further clarifying intervention targets.

As another crucial pathway in the COM-B model, capability plays a significant role in promoting PA behavior. In previous literature, the assessment of PA capability in older adults with SCD or older adults with dementia/MCI has often used indicators like activities of daily living (ADL) (20), which focus on assessing an older individual’s self-care ability or the severity of their illness, rather than specifically targeting indicators related to PA ability. Physical literacy (21) refers to an individual’s ability to acquire and understand information about PA and health promotion throughout their life, utilizing this information to promote participation in physical activities and maintain comprehensive health. Although physical literacy research has predominantly focused on adolescents and adults (22), its significance in promoting healthy aging is substantial, embodying a holistic approach to physical and mental well-being. Physical literacy spans an individual’s entire life, allowing people of different ages to develop the knowledge, skills, and attitudes needed to participate in various activities. Thus, compared to one-dimensional indicators such as ADL, physical literacy better reflects the multidimensional aspects of PA ability in older adults, including knowledge, skills, attitudes, and more. Therefore, this study introduces physical literacy as a measure of capability in older adults with SCD for the first time, hypothesizing that higher levels of physical literacy correspond to stronger motivation for engagement in PA behaviors, leading to the realization of PA.

**Figure 1:**
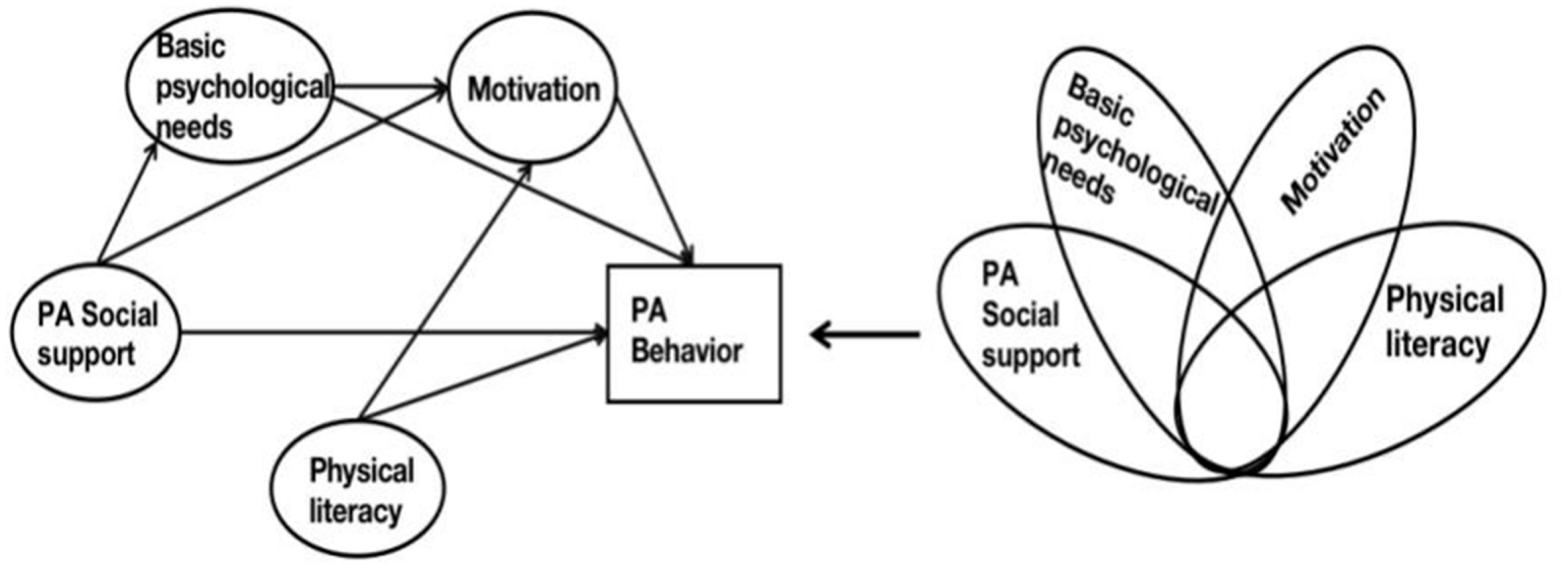
Conceptual model. On the left is the conceptual model of SEM, and on the right is the conceptual model of fsQCA.

Current research has predominantly explored the influencing factors of PA behavior in older adults with dementia/MCI (23–25), without delving into the intricate internal operating mechanisms between these factors, failing to provide guidance on the mechanisms of action for influencing factors on PA behavior in older adults with SCD. Given that SCD represents a crucial secondary prevention stage in the progression toward dementia/MCI, promoting PA in this context is highly significant, and understanding the internal mechanisms of influencing factors on PA behavior is fundamental. This study seeks to investigate the operational mechanisms of influencing factors on PA behavior in older adults with SCD, aiming to identify a practical and effective intervention approach to inform the formation and promotion of lifelong PA behavior in this population. Therefore, the first focus of this study is on the internal operating mechanisms of PA social support on PA behavior in older adults with SCD. This study hypothesizes that PA social support positively predicts PA behavior in older adults with SCD, with the degree of basic psychological needs and motivation having a progressively linked mediating effect in this influence. The second focus is to explore the internal operating mechanisms of physical literacy on PA behavior in older adults with SCD. This study hypothesizes that physical literacy positively predicts PA behavior in older adults with SCD, with motivation serving as a mediating factor in this influence (Figure 1). Furthermore, to better reveal the causal configurations leading to physical activity behavior in older adults with SCD, we employed fsQCA analysis (26). Firstly, structural equation modeling (SEM) is proficient at scrutinizing linear associations among variables, highlighting straightforward and symmetrical connections between antecedents and outcomes. However, it falls short in illustrating intricate multifactorial and simultaneous causal relationships. Secondly, fsQCA proves beneficial for scrutinizing the consolidation of relationships and executing more comprehensive pathways. Thirdly, social phenomena are complex, and causal mechanisms may fluctuate under diverse conditions. Therefore, it is essential to systematically identify causal logics to better explain complex social phenomena. The combined analysis approach of SEM and fsQCA allows for a more comprehensive exploration of the mechanisms underlying the formation of physical activity behavior in older adult SCD individuals, providing practical insights for promoting physical activity in this population. The theoretical hypothesis diagram for this study is provided below (Figure 1).

## Methods

### Study design

The study was an observational cross-sectional study. The Strengthening the Reporting of Observational Studies in Epidemiology (STROBE) statement was used to report this observational study.

### Setting and sampling

This research was carried out in a county town situated in Southeastern Fujian Province characterized by a significant migration of its young and middle-aged labor force and an increasing aging population during the period of July to November 2023. In the latest census of the county town, the population aged 60 and above was 82,859, constituting 19.61% of the total population. Our research team contacted the directors of residents’ committees in 15 communities within the town, seeking their cooperation in distributing notices about participating in the questionnaire survey. Special senior activity rooms were set up in respective community committees to facilitate older adults in completing the questionnaires. Eventually, directors from 12 communities agreed to collaborate with our research. As a token of appreciation, each older participant completing the SCD screening received a complimentary blood pressure measurement service and a small gift, specifically a pack of tissues. Furthermore, eligible older individuals completing the questionnaire received additional compensation in the form of a red envelope worth 50 RMB. Inclusion criteria for participants were: (1) age 60 years or older; (2) meeting the criteria for subjective cognitive decline (i.e., a score greater than 5 on the Subjective Cognitive Decline 9-item (SCD-9) questionnaire (27) and a Mini-Mental State Examination scale (28) score greater than or equal to 27); and (3) the ability to communicate in Mandarin or a local dialect. Exclusion criteria included: (1) participation in psychosocial interventions during the study period; (2) severe disability; and (3) severe liver or kidney disease or any known malignancy.

The sample size for this study was determined based on established guidelines for structural equation models (29), recognizing the need for a minimum of 10–20 participants per observed variable for structural equation analysis. In this study, we designated 20 participants for each of the 12 observed variables, including age, gender, marital status, place of residence, educational attainment, current smoking status, current alcohol consumption, personal monthly income, physical literacy, PA social support, basic psychological needs, and motivation. Accounting for a 10% rate of invalid questionnaires, the final minimum sample size was set at 267 participants. Throughout the survey process, 391 older adults who self-reported memory decline using the SCD-9 questionnaire were initially screened, with 323 meeting the criteria for SCD. Ultimately, 303 older adults with SCD completed the questionnaire survey, and no invalid questionnaires were identified, resulting in a response rate of 93.8%. Participants spent approximately 20–30 min completing the questionnaire. The ethical approval for this study were obtained from the institutional review board of Shanxi Medical University. Prior to participation, informed consent was obtained from all participants.

### Measures

#### Socio-demographic characteristics

The general information form aims to collect socio-demographic data, including age, gender, marital status (married/living with partner or unmarried/widowed), place of dwelling (rural/urban), educational attainment (elementary school or below, middle school, high school or above), current smoking status (yes/no), current alcohol consumption (yes/no), and personal monthly income.

#### Physical activity behavior

The physical activity behavior of older adults with SCD were assessed from the perspectives of intensity, duration, and frequency, utilizing the Physical Activity Rating Scale-3 (PARS-3) developed by Liang (30). This scale employs a Likert 5-point scoring method, ranging from 1 (completely non-compliant) to 5 (completely compliant). The physical activity score is calculated as the product of intensity, duration minus one, and frequency. The score for physical activity ranges from 0 to 100, with 100 being the highest possible score. This scale has been widely applied across various age groups in China (31).

#### Physical literacy

We utilized the Perceived Physical Literacy Instrument (simplified Chinese version) to assess the physical literacy of older adults with SCD. Originally developed by scholar Whitehead (32), the Perceived Physical Literacy Instrument was later adapted for Chinese culture by Ma et al. (33) The instrument consists of three dimensions (Confidence and physical competence, Motivation, Interaction with the environment) and eight items. The instrument employs a 5-point Likert scale for scoring, with responses ranging from 1 (strongly disagree) to 5 (strongly agree). The total score ranges from 8 to 40. The higher the score, the higher the level of physical literacy. Cronbach’s alpha for reliability ranges from 0.79 to 0.83 (33). Prior to the formal survey, our research team conducted a validity test of the instrument among the older adult population, confirming its high validity in this demographic.

#### Basic psychological needs

In this study, we employed the Exercise Psychological Need Satisfaction Scale (PNSE), originally developed by Wilson et al. (34). Subsequently, Liu Lei and colleagues (35) conducted a validation study on this scale among older adult residents in Chinese nursing homes, establishing its reliability and validity. Consequently, the PNSE can be utilized to assess the psychological need satisfaction related to exercise among older adults in Chinese nursing homes. The PNSE comprises three dimensions: autonomy, competence, and relatedness needs, each consisting of six items, resulting in a total of 18 items. The scoring system for the scale utilized a 7-point rating, ranging from “completely disagree” to “completely agree,” with scores assigned between 1 and 7, respectively. Higher scores on the scale indicate an increased level of satisfaction with fundamental psychological needs.

#### Physical activity social support

The variable of “PA social support” was measured using the Social Support Scale developed by Taiwanese scholar Hong Huang-Jia et al. (36) as a Chinese version. It was later optimized by mainland Chinese scholar Duan Mengshuang (37) to suit the measurement of social support for PA among the older population in mainland China (38). This scale has since been validated in several dissertations by mainland scholars and has shown good reliability and validity. This scale is specifically designed to assess the support received by older adults for their physical activity behaviors. It includes four dimensions: family support, friend support, informational support, and instrumental support, comprising a total of 17 assessment items. It effectively evaluates the physical and social support required for the PA behaviors of the older adult. In this scale, which served as an independent variable, a 5-point Likert scale was employed. Responses ranged from “completely disagree” to “completely agree,” with scores ranging from 1 to 5. The total score ranges from 17 to 105, with higher scores indicate a higher level of PA social support.

#### Motivation

To investigate PA motivation in older adults with SCD, all participants completed the Shorten Motives for Physical Activity Measure-Revised (Shorten MPAM-R) questionnaire. The Motives for Physical Activity Measure-Revised (MPAM-R) was originally developed by Richard et al. in 1997 (39), comprising 30 items. To better suit the Chinese context, Chinese scholar Chen Shanping introduced this scale and conducted a simplified revision, resulting in the Shorten MPAM-R (40). The Shorten MPAM-R employs a 5-point Likert scale, ranging from 1 (lowest motivation) to 5 (highest motivation), to assess the extent to which personal motives. It has 15 items and includes 5 subscales: fun motivation (3 items), ability motivation (3 items), appearance motivation (3 items), health motivation (3 items), and social motivation (3 items). The total score ranges from 5 to 75, with higher scores indicating greater motivation and lower scores indicating lower motivation.

### Data analysis

The data were analyzed using SPSS 27.0 and AMOS 23.0 (IBM Corp., Armonk, NY, USA). All variables in this study adhere to a normal distribution. Descriptive statistics for continuous data were computed using means and standard deviations (SD), while categorical variables were summarized with frequencies and proportions. In the univariate analysis, only age showed statistical significance (*p* < 0.05) and was included as a control variable in the subsequent SEM analysis. The total and specific indirect effects were computed using bootstrapping with 5,000 samples. Model fit indices for path analysis included the *χ*^2^ test, root mean square error of approximation (RMSEA) ≤ 0.08, comparative fit index (CFI) ≥ 0.95, and Tucker Lewis index (TLI) ≥ 0.95 (41).

Fuzzy-set qualitative comparative analysis is a type of Qualitative Comparative Analysis (QCA) method, which reveals asymmetric and complex causal relationships between antecedent variables and outcomes by constructing fuzzy set data (42). Given its suitability for small to medium-sized sample sizes, we followed the approach of previous researchers (43) and randomly selected half of the total sample of 303 cases (151 cases) for fsQCA data analysis. fsQCA presents results in the form of simple solutions, intermediate solutions, and complex solutions, and evaluates the degree of membership and explanatory power based on two parameters: consistency and coverage. The values of consistency and coverage range from 0 to 1. Acceptance is generally considered when the consistency level exceeds 0.75, with higher values indicating a higher level of explanatory power (44). In the fsQCA antecedent variables, to further explore the specific dimensions of PA social support in relation to the outcome variable, we included four dimensions of PA social support (family support, friend support, informational support, and instrumental support) as four antecedent variables, along with physical literacy, basic psychological needs, and motivation, totaling seven antecedent variables. This quantity is considered ideal for conducting fsQCA.

## Results

### Demographic information and correlation analysis

In this study, participants had an average age of 67.8 ± 5.1; among them, 56.8% were female participants. 78.9% of participants were married or living with a partner, and the majority of participants (69.3%) living in rural areas. Approximately half of the participants (50.5%) had an educational level of junior high school or above. 36.3% of participants smoked, and 28.1% consumed alcohol. Finally, nearly half of the participants (46.5%) reported a monthly personal income in the range of 2000–3,000 yuan (Table 1). As shown in Supplementary Table S1, PA behavior was positively correlated with PA social support (r = 0.425; *p* < 0.01), physical literacy (r = 0.392; *p* < 0.01), basic psychological needs (r = 0.447; *p* < 0.01), and motivation (r = 0.434; *p* < 0.01), with statistically significant differences.

**Table 1:** Characteristics of participants (*n* = 303).

Variables	Frequency (%)
Gender	
Male	172 (56.8)
Female	131 (43.2)
Marital status	
Married/Living with partner	239 (78.9)
Unmarried/Widowed	64 (21.1)
Place of living	
Rural area	210 (69.3)
Urban area	93 (30.7)
Educational attainment	
Primary school and below	150 (49.5)
Middle school	103 (34.0)
High school and above	50 (16.5)
Current smoker	
No	193 (63.7)
Yes	110 (36.3)
Current drinking	
No	218 (71.9)
Yes	85 (28.1)
Personal monthly income (RMB)	
≤1,000	49 (16.2)
1,000 < RMB ≤ 2000	52 (17.2)
2,000 < RMB ≤ 3,000	141 (46.5)
>3,000	61 (20.1)

### Structural equation modeling results

**Figure 2:**
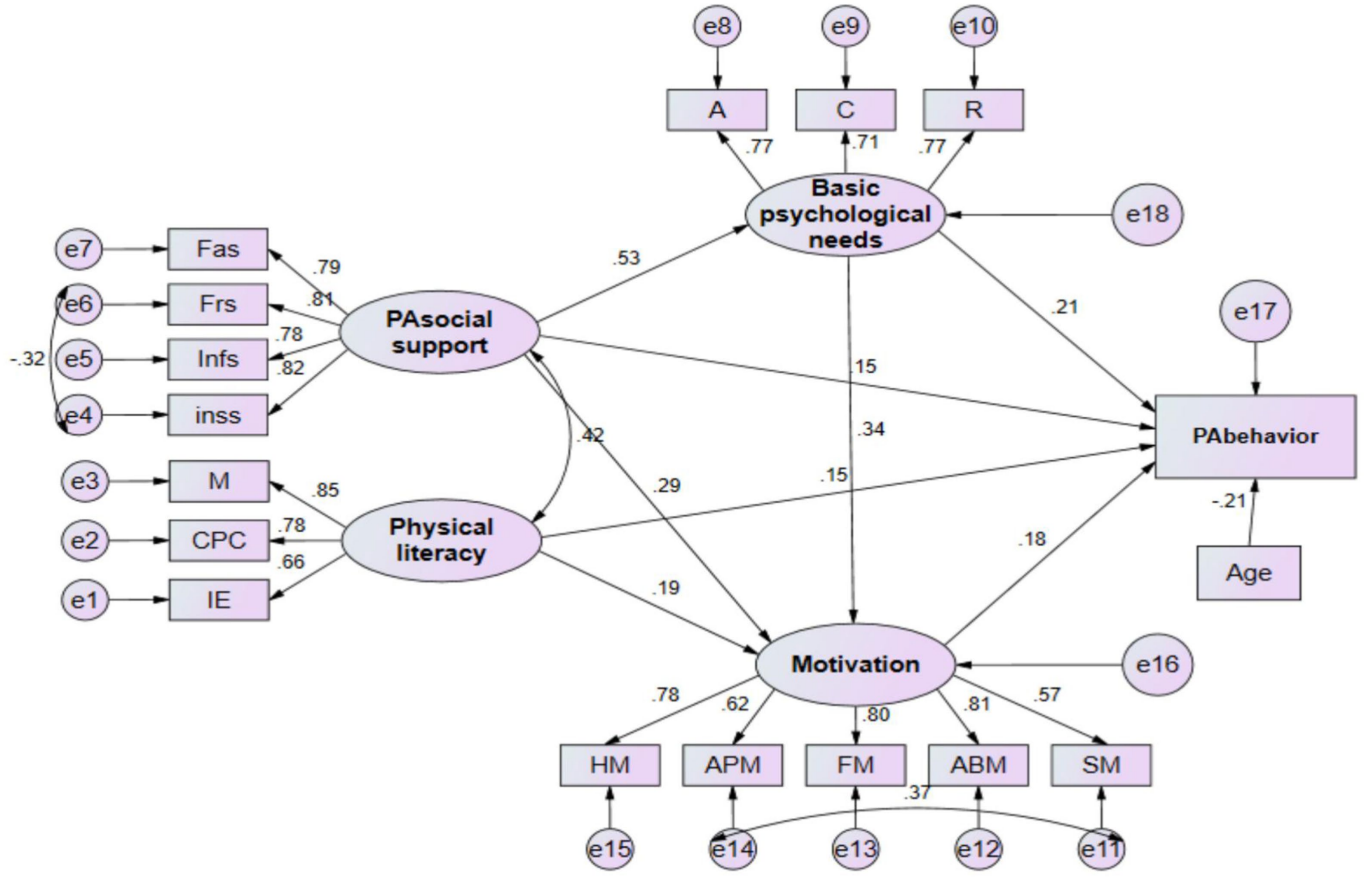
Factors influencing physical activity behavior in older adults with subjective cognitive decline. All relationships are significant. A, autonomy, C, competence; R, relatedness needs; Fas, family support; Frs, friend support; Infs, informational support; Inss, instrumental support; M, motivation; CPC, confidence and physical competence; IE, Interaction with the environment; HM, health motivation; APM, appearance motivation; FM, fun motivation; ABM, ability motivation; SM, social motivation. Model fit indices: x2/df = 2.461, RMSEA = 0.070, GFI 0.909, CF1931, IFI 0.932, TLI = 0.914, AGFI = 0.872.

Before conducting SEM, the balance method (45) was employed to bundle the measurement indicators of PA social support, Physical literacy, Basic psychological needs, and motivation into four latent variables, each comprising 4, 3, 3, and 5 measurement indicators, respectively. PA behavior served as the observed variable. Based on the theoretical frameworks of SDT and the COM-B model, the AMOS 26.0 software was used to construct an intermediate hypothesis model (Figure 2). The entire hypothesis model was tested using maximum likelihood estimation, revealing that the data in the selected predictive model conformed to univariate normal distribution. However, the overall predictive model did not adhere to multivariate normality. Correction was applied using the MI index method (46). After the correction, all fit indices of the model met the standard range, namely *χ*^2^/df = 2.461, RMSEA = 0.070, GFI = 0.909, CFI = 931, IFI = 0.932, TLI = 0.914, AGFI = 0.872. This indicates that the structural equation data fit well with the selected model.

From Figure 2 and Table 2, it can be observed that: (1) Regarding basic psychological needs, the impact of PA social support on basic psychological needs reached a significant level, with a standardized regression coefficient of 0.534 (*p* < 0.001); (2) In terms of motivation, the impact of basic psychological needs, PA social support, and physical literacy on motivation all reached significant levels, with standardized regression coefficients of 0.338 (*p* < 0.001), 0.290 (*p* < 0.001), and 0.192 (*p* = 0.004), respectively; (3) Concerning PA behavior, PA social support, physical literacy, basic psychological needs, and motivation all significantly influenced PA behavior, with standardized regression coefficients of 0.209 (*p* = 0.005), 0.150 (*p* = 0.039), 0.176 (*p* = 0.020), and 0.149 (*p* = 0.017), respectively.

**Table 2:** Path coefficients between variables (*n* = 303).

Path			β	b	S.E.	C.R.	*p*-value
Basic psychological needs	←	PA social support	0.534	0.77	0.101	7.622	<0.001
Motivation	←	Basic psychological needs	0.338	0.173	0.041	4.231	<0.001
Motivation	←	PA social support	0.290	0.215	0.059	3.645	<0.001
Motivation	←	Physical literacy	0.192	0.118	0.041	2.885	0.004
PA behavior	←	Basic psychological needs	0.209	2.779	0.997	2.788	0.005
PA behavior	←	PA social support	0.150	2.886	1.400	2.061	0.039
PA behavior	←	Motivation	0.176	4.562	1.955	2.334	0.020
PA behavior	←	Physical literacy	0.149	2.368	0.995	2.380	0.017

PA, physical activity; S.E., standard error; C.R., critical ratio.

The indirect effects between variables in the model were examined using the bias-corrected bootstrap estimation method with a 95% confidence interval (*N* = 5,000). From Table 3 and Figure 2, it can be observed that, after controlling for the meaningful covariate of age: Firstly, PA social support has a significant indirect effect on PA behavior through three specific paths: (1) PA social support → Basic psychological needs → PA behavior (*β* = 0.262; 95% CI = 0.137 to 0.382; *p* < 0.001); (2) PA social support → Motivation → PA behavior (β = 0.194; 95% CI = 0.074 to 0.307; *p* = 0.001); and (3) PA social support → Basic psychological needs → Motivation → PA behavior (β = 0.480; 95% CI = 0.302 to 0.638; *p* < 0.001). The percentage of total effects accounted for by these three paths is 42.4, 22.2, and 68.5%, respectively. Additionally, physical literacy also has a significant indirect effect on PA behavior through the path: Physical literacy → Motivation → PA behavior (β = 0.192; 95% CI = 0.031 to 0.351; *p* < 0.001), with the indirect effect accounting for 15.1% of the total effect.

**Table 3:** Mediation analysis (*n* = 303).

Path	Effect decomposition	Point estimate	S.E.	Lower 95%CI	Higher 95%CI	*p*-value
PA social support → Basic psychological needs → PA behavior	Mediation effect	0.111	0.047	0.027	0.213	0.008
	Direct effect	0.150	0.057	0.035	0.258	0.011
	Total effect	0.262	0.062	0.137	0.382	0.000
PA social support → Motivation → PA behavior	Mediation effect	0.043	0.022	0.008	0.097	0.013
	Direct effect	0.150	0.057	0.035	0.258	0.011
	Total effect	0.194	0.060	0.074	0.307	0.001
PA social support → Basic psychological needs → Motivation → PA behavior	Mediation effect	0.329	0.076	0.175	0.471	0.001
	Direct effect	0.150	0.057	0.035	0.258	0.011
	Total effect	0.480	0.086	0.302	0.638	0.000
Physical literacy → Motivation → PA behavior	Mediation effect	0.029	0.017	0.005	0.077	0.011
	Direct effect	0.149	0.062	0.024	0.267	0.021
	Total effect	0.192	0.081	0.031	0.351	0.020

PA, physical activity; S.E., standard error; CI, confidence interval.

### Results of fsQCA

Table 4 summarizes the four paths for promoting PA behavior according to the format outlined by Peer (30). In predicting PA behavior, four paths were observed that explained 40.9% of the cases (Overall Consistency = 0.838; Overall Coverage = 0.409).

**Table 4:** High-level physical activity configurations.

Variables		Path 1	Path 2	Path 3	Path 4
PA social support	Family support	●	●	●	●
	Friend support	⊗		●	●
	Informational support		⊗	●	●
	Instrumental support	⊗	●	●	⊗
Basic psychological needs		●	●		⊗
Motivation		⊗	●	●	●
Physical literacy		●	●	●	⊗
Raw coverage		0.103	0.132	0.262	0.052
Unique coverage		0.061	0.046	0.185	0.020
Consistency		0.881	0.854	0.847	0.831
Solution coverage		0.409			
Solution consistency		0.838			

Black circles indicate the presence of a condition, and circles with “X” indicate the absence of a condition. Large circles indicate core conditions and small circles indicate peripheral conditions. Blank spaces indicate “do not care.” Overall, the solution coverage is the total coverage for all configurations.

Paths 1 and 2: Path 1 indicates that a combination of basic psychological needs and physical literacy as core conditions, with family support as a moderating condition, can effectively promote PA behavior. This path can account for 10.3% of individuals engaging in high levels of PA. Path 2, on the other hand, suggests that a combination of five elements—family support, instrumental support, basic psychological needs, motivation, and physical literacy—as core conditions within social support can effectively promote PA behavior. This path can explain 13.2% of individuals engaging in high levels of PA. In other words, for older adults with only one or two forms of social support, in addition to increasing their motivation and physical literacy, meeting their exercise-related psychological needs is essential to effectively promote their PA behavior.

Paths 3 and 4: Path 3 indicates that a combination of four core conditions—friend support, instrumental support, motivation, and physical literacy—with family support and informational support as moderating conditions can promote PA behavior. This path can explain 26.2% of the occurrence of individuals’ PA behavior and is a major pathway. Path 4, on the other hand, suggests that a combination of family support and motivation as core conditions, with friend support and informational support as moderating conditions, can effectively promote physical activity behavior. This path can explain 5.2% of individuals engaging in high levels of PA. In summary, for older adults with higher levels of social support across multiple dimensions, promoting their motivation for PA and increasing their level of physical literacy can effectively enhance their PA behavior.

The fsQCA method is highly applicable to the issue of causal asymmetry, where the configuration of antecedent conditions for a certain outcome to occur or not is not entirely opposite. To elucidate the driving mechanism behind PA behavior in older adults with SCD, a further analysis was conducted to examine the configurations of antecedent conditions hindering PA behavior. According to Table 5, Path A indicates that when all factors are at low levels, PA levels in older adults with SCD are low. This pathway has the highest coverage, reaching 28.2%. It suggests that the low PA levels in older adults with SCD result from the combined effects of multiple factors. Furthermore, instrumental support is a common core missing condition across the three pathways, indicating that the absence of instrumental support is a necessary condition leading to low PA levels in older adults with SCD.

**Table 5:** Low-level physical activity configurations.

Variables		Path A	Path B	Path C
PA social support	Family support	⊗	●	⊗
	Friend support	⊗	⊗	●
	Informational support	⊗	⊗	●
	Instrumental support	⊗	⊗	⊗
Basic psychological needs		⊗	⊗	⊗
Motivation		⊗	⊗	⊗
Physical literacy		⊗	●	⊗
Raw coverage		0.282	0.063	0.062
Unique coverage		0.244	0.027	0.045
Consistency		0.872	0.907	0.848
Solution coverage		0.356		
Solution consistency		0.872		

Black circles indicate the presence of a condition, and circles with “X” indicate the absence of a condition. Large circles indicate core conditions and small circles indicate peripheral conditions. Blank spaces indicate “do not care.” Overall, the solution coverage is the total coverage for all configurations.

## Discussion

The integration of SEM and fsQCA in this study yielded several key findings. Firstly, the theoretical model, combining the SDT and COM-B framework, effectively explained and predicted the interrelationships among variables. A previous study (47) has indicated that these two theories remain relatively underexplored in research on promoting physical activity among the older adults. Our findings further contribute to this area. Secondly, perceived social support for PA emerged as crucial in promoting PA behavior in older adults with SCD, with basic psychological needs and motivation playing vital chain mediating roles. Additionally, higher levels of physical literacy not only directly promoted PA behavior but also positively influenced PA behavior through enhanced motivation. Thirdly, the multifactorial nature of PA behavior in older adults with SCD was evident. Lastly, for those with high levels of PA social support across multiple dimensions, elevating physical literacy and PA motivation effectively promoted PA behavior. Conversely, individuals with fewer dimensions of high-level PA social support required attention to meeting basic psychological needs, in addition to the previously mentioned variables, to foster PA behavior in older adults with SCD.

Social support for PA plays a positively predictive role in PA behavior in older adults with SCD, demonstrating a significant facilitating effect. As previously discussed in the literature (11), physical resources and social influences are identified as the two most crucial factors influencing MCI/dementia in older adults. In this study, PA social support encompasses four dimensions: family support and friend support (social influences) as well as informational support and instrumental support (physical resources). It serves as a comprehensive indicator for evaluating social support for physical activity in older adults with SCD, addressing a broader range compared to previous studies that often focused more on support from family or friends while overlooking informational and instrumental support (48, 49). Perceiving PA social support is conducive to older adults with SCD engaging in PA with increased positive emotional involvement, leading to stable, intrinsic, and enduring impacts on their active participation. A qualitative study (50) has also indicated that some older individuals prioritize emotional support provided by personal trainers or exercising with peers during exercise programs, meeting their psychological needs and enhancing their motivation for exercise. The fit of the model results suggests that perceiving PA social support is essential for promoting physical activity behavior in older adults with SCD. Social support creates conditions for the formation of motivation for PA in this population, making it an effective factor for predicting and influencing PA behavior in older adults with SCD. Specifically, family support and friend support enhance the emotional connection and participation motivation of older adults with SCD through providing emotional encouragement and social interaction. These social interactions not only offer psychological comfort but also enhance their sense of belonging and responsibility by participating in PA activities together, thereby improving the persistence of their exercise. Furthermore, informational support and instrumental support reduce their barriers to engaging in PA by providing practical exercise knowledge, guidance, and physical resources such as exercise equipment and venues, making it easier for them to initiate and maintain an exercise regimen.

The fit results of the influencing factors model for PA behavior in older adults with SCD further clarify the underlying mechanism through which perceived social support for PA affects their behavior. It suggests that the impact of social support on motivation and PA behavior in older adults with SCD is contingent upon the support being perceived by these individuals and meeting their basic psychological needs. Autonomy, competence, and relatedness are three universally recognized basic psychological needs, and prior research (51, 52) suggests that when individuals’ psychological needs for autonomy, competence, and relatedness are satisfied, they are more likely to initiate and sustain a wide range of behaviors. Additionally, a previous study (53) indicates a positive association between the satisfaction of these psychological needs and exercise adherence in older adults. Furthermore, our research reveals that fulfilling basic psychological needs directly mediates motivation and, consequently, has a direct intermediary role in influencing PA behavior. This aligns with previous research findings (54) that establish a close correlation between motivation and autonomy within the basic psychological needs. In our fsQCA results, we also find that the unsatisfaction of basic psychological needs is a necessary factor in all paths leading to low-level PA combinations. This suggests that fulfilling these needs is essential for individuals to perceive behavior as self-determined and to have the motivation to engage in PA. Conversely, when these needs are not satisfied, the motivation for engaging in PA diminishes (55). Healthcare professionals working with older adults with SCD should consider the possibility of promoting PA by enhancing psychological needs satisfaction through increased autonomy, competence, and/or relatedness.

Physical literacy has emerged as a prominent field in sports science research in recent years (21). Individuals with physical literacy continuously acquire motivation and abilities for development, enabling them to learn, analyze, apply, and demonstrate various forms of PA. They can engage in a variety of sports activities in different environments, showcasing competence and confidence, making healthy and positive choices, and demonstrating respect for themselves, others, and the environment. Consequently, physical literacy aligns to some extent with the concept of health literacy, but as a specific concept emphasizing physical activity, it better explains the comprehensive ability and literacy of individuals engaging in physical activity. In this study, we have, for the first time, confirmed the positive impact of physical literacy on PA behavior in older adults with SCD, not only through direct influence but also by enhancing motivation for PA. Previous research (56) has also indicated that individuals with physical literacy cognitively understand their psychological resources in declarative and procedural forms and can utilize these resources to stimulate their motivation for engaging in PA.

According to the results of fsQCA, we also found that older adults with SCD exhibiting high levels of PA display characteristics of multiple and diverse pathways. Social support for PA, basic psychological needs, physical literacy, and motivation are not individually necessary conditions leading to PA behavior in older adults with SCD. Instead, these factors collectively interact as antecedent conditions, highlighting the complexity of PA behavior in this population and affirming the rationale behind the research framework used. Furthermore, for older adults with SCD experiencing limited dimensions of social support, it is essential not only to focus on their physical literacy and motivation but also to address their basic psychological needs for PA. This suggests a significant relationship between perceived social support and basic psychological needs. When the dimensions of social support sources are limited, older adults with SCD may have relatively insufficient fulfillment of their basic psychological needs. Prior research also indicates (49) that singular social support from friends or family may not consistently positively influence the PA behavior of older individuals. Therefore, when faced with limited diversity in social support sources, interveners should strive to enhance the physical literacy, motivation, and basic psychological needs of older adults with SCD.

## Limitations

While this study has made positive explorations into the influencing factors of PA behavior in older adults with SCD, there are still some limitations. Firstly, the sample in this study is limited to a township in southeastern Fujian Province, which may introduce regional restrictions. This could affect the generalizability of the results. It is recommended that future research includes plans to expand the sample to cover more regions and different cultural backgrounds. Secondly, this study employs a cross-sectional design, which can only describe correlations between variables and cannot infer causality. Although SEM and fsQCA reveal complex relationships, they cannot replace longitudinal studies for causal inference. Future research may consider using a longitudinal design to track changes in PA behavior and influencing factors among older adults with SCD. Additionally, due to the limitations in the number of antecedent variables in fsQCA, the analysis of some other variables’ finer dimensions (such as basic psychological needs, physical literacy, etc.) may not be well-addressed, potentially missing some combinations of pathways. Future research can fine-tune the selection of antecedent variables. Thirdly, although configurational analysis can complement the shortcomings of structural equation modeling, it still falls short in explaining the “why” and “how” of PA behavior. Subsequent studies can integrate configurational analysis with case studies to further explore relevant issues related to the influencing factors of PA behavior in older adults with SCD, enhancing the explanatory power of the research. Lastly, the questionnaire relies on participants’ self-reports, which may introduce recall bias and social desirability effects. Future research could incorporate objective measurements, such as using wearable devices to record PA data, to enhance data accuracy.

## Conclusion

The theoretical model derived from the SDT and the COM-B model effectively predicts and promotes PA behavior in older adults with SCD. Perceived social support for PA is pivotal in encouraging PA in this group. Basic psychological needs and motivation play crucial roles as chain mediators. Higher levels of physical literacy not only directly promote physical activity but also enhance motivation, further encouraging PA. The emergence of high-level PA results from the combined effects of social support, basic psychological needs, motivation, and physical literacy. Even in older adults with SCD where multiple dimensions of PA social support are at high levels (three dimensions or more), it is still necessary to significantly improve their physical literacy and PA motivation to promote physical activity behavior. For individuals with fewer dimensions of PA social support at high levels (two dimensions or fewer), in addition to focusing on the above two variables, meeting their basic psychological needs is essential to effectively promote their PA behavior.

## Data Availability

The raw data supporting the conclusions of this article will be made available by the authors, without undue reservation.
