# Supplementary material for: Factors influencing physical activity behavior in older adults with subjective cognitive decline: an empirical study using SEM and fsQCA methods
Source: Front Public Health. 2024 Oct 17;12:1409614. doi: 10.3389/fpubh.2024.1409614 (PMC11526387; doi:10.3389/fpubh.2024.1409614)
Supplement: Supplementary file 1 [file Table_1.pdf]

Table S1 Correlations among main variables (n =303)

	PA social support	Physical literacy	Basic psychological needs	Motivation	PA behavior
PA social support	1				
Physical literacy	.355**	1			
Basic psychological needs	.426**	.489**	1		
Motivation	.464**	.392**	.479**	1	
PA behavior	.425**	.392**	.447**	.434**	1
Mean	3.003	2.512	3.429	3.348	15.208
SD	0.870	1.078	1.004	0.814	14.770

Abbreviation: PA, Physical activity; SD, standard deviation. **, P < 0.01.

Table S2 Necessity analysis for physical activity behavior

Variable	consistency	coverage
Family support	0.685	0.703
~Family support	0.442	0.474
Friend support	0.632	0.680
~Friend support	0.474	0.483
Informational support	0.650	0.667
~Informational support	0.461	0.492
Instrumental support	0.655	0.661
~Instrumental support	0.460	0.500
Basic psychological needs	0.618	0.651
~Basic psychological needs	0.498	0.519
Motivation	0.641	0.642
~Motivation	0.464	0.509
Physical literacy	0.602	0.664
~Physical literacy	0.506	0.504
